# The hollow fibre assay as a model for *in vivo* pharmacodynamics of fluoropyrimidines in colon cancer cells

**DOI:** 10.1038/sj.bjc.6603507

**Published:** 2006-12-19

**Authors:** O H Temmink, H-J Prins, E van Gelderop, G J Peters

**Affiliations:** 1 Department of Medical Oncology, VU University Medical Center, Amsterdam, The Netherlands; 2 Clinical and Experimental Animal Laboratory, Faculty of Medicine, Vrije Universiteit, Amsterdam, The Netherlands

**Keywords:** antimetabolites, colon cancer, fluoropyrimidines, hollow fibre assay, *in vivo* pharmacodynamics, TAS-102

## Abstract

The Hollow Fibre Assay (HFA) is usually applied as an early *in vivo* model for anti-cancer drug screening, but is potentially an excellent model for short-term *in vivo* pharmacodynamic studies. We used the model to study the *in vivo* role of thymidine phosphorylase/platelet-derived endothelial cell growth factor (TP/PD-ECGF) in the cytotoxicity and pharmacodynamics of TAS-102 in colon cancer cells. TAS-102 is a new oral drug formulation, which is composed of trifluorothymidine (TFT) and thymidine phosphorylase inhibitor (TPI), which prevents TFT degradation. We compared the activity with Xeloda (capecitabine), which is activated by TP into 5FU. Hollow fibres filled with human Colo320 or Colo320TP1 colorectal cancer cells with deficient or high TP expression, respectively, were implanted subcutaneously (s.c.) at both flanks of BALB/c mice. The mice were treated orally over 5 days with TAS-102, TFT alone, 5′DFUR±TPI or capecitabine at their maximum tolerated dose (MTD). The cells were retrieved from the fibres and assayed for growth (MTT assay), cell cycle distribution (flow cytometry) and apoptosis induction (FragEL method). TAS-102 induced considerable growth inhibition (50%, *P*<0.01) to both cell lines, which was completely abolished in the absence of TPI. Capecitabine and its metabolite 5′DFUR reduced proliferation of Colo320TP1 cells in the fibres significantly (down to 25–40%), but much less in Colo320 cells, whereas addition of TPI reduced the effect of 5′DFUR, although not completely. These differences in cytotoxic effects were reflected in the pharmacodynamic evaluation. TAS-102 induced a G2M-phase arrest (from 25 to 40%) and apoptosis (>8-fold), which was more pronounced in Colo320 than in Colo320TP1. Again, omission of TPI neutralised the effect of TAS-102. Similarly, 5′DFUR and capecitabine induced a significant G2M-phase arrest (up to 45%) in the Colo320TP1 cell line, but less pronounced in the parental Colo320. Addition of TPI to 5′DFUR reduced this effect to control levels. Also induction of apoptosis was reduced in the presence of TPI. The data demonstrated that the HFA is excellently suited for studying short-term pharmacodynamic effects of fluoropyrimidines *in vivo*. TAS-102 is only effective in inducing cytotoxicity when systemic TPI is present, but acts against both low and high TP expressing colon cancer cells, while 5′DFUR needs cellular TP to exert significant activity.

Several preclinical *in vitro* and *in vivo* tumour models are being used for the screening of potential anticancer agents. Currently, the primary focus of the National Cancer Institute (NCI) is on the 60-tumour cell line panel. This system is based on selection of cell lines from major disease types (Venditti *et al*, 1984) in which the nonclonogenic microculture tetrazolium (MTT) assay or sulphorhodamine B (SRB) assay were selected to determine cytotoxicity of the tumour cells (Alley *et al*, 1988; Skehan *et al*, 1990; Keepers *et al*, 1991). When positive (e.g. a unique profile in the COMPARE program), the compounds are subjected to a secondary screening with several tumour xenograft models, the Hollow Fibre Assay (HFA) (Hollingshead *et al*, 1995; Hollingshead *et al*, 1999; Suggitt and Bibby, 2005), the human tumour stem cell (HTSC) assay (Von Hoff *et al*, 1980; Moon *et al*, 1981), and other screening model systems including various types of clonogenic assays (Fiebig *et al*, 1987; Fiebig *et al*, 2004). In most *in vivo* models and multicellular *in vitro* models mechanistic studies are not included, because they are cumbersome, while *in vivo* reproducible retrieval of the tumours is often a limitation. However, it is evident that *in vivo* models and multicellular systems are closer to a clinical situation than *in vitro* models (Suggitt and Bibby, 2005).

The *in vivo* HFA animal model (Mi *et al*, 2002; Decker *et al*, 2004), optimised by Hollingshead *et al* (1995) (Hollingshead *et al*, 1995), uses semipermeable biocompatible fibres that are filled with cancer cells, heat-sealed and implanted surgically (s.c. or i.p.) in mice or rats, which can be treated with chemotherapeutics. Many different cell lines from different tissue origins and cellular characteristics can be encapsulated within the fibres, providing a cost-effective screening method. Most active agents screened positive in a *in vitro* multicell line screening assay or indicated xenograft-positive will not be bypassed in the HFA (Hollingshead *et al*, 1999; Johnson *et al*, 2001; Decker *et al*, 2004). In the HFA, cytotoxicity is determined using a modified MTT assay, taken into account the *in vivo* parameters, such as pharmacokinetics and drug transport/pH/pO_2_ at tumour site (Phillips *et al*, 1990; Jonsson *et al*, 2000). Additionally, several pharmacodynamic end points can also be investigated, such as DNA damage induction, apoptosis or cell cycle analysis (Hall *et al*, 2000; Leong *et al*, 2004; Suggitt *et al*, 2004). This methodology also saves time (assay <2 weeks) and the number of animals used in the experiments.

The novel antitumour drug formulation TAS-102 (Emura *et al*, 2004c; Tsuchiya *et al*, 2004; Emura *et al*, 2005) combines the cytotoxic agent 5-trifluoro-2′-deoxythymidine (TFT) with an inhibitor of the angiogenic thymidine phosphorylase/platelet-derived endothelial cell growth factor (TP/PD-ECGF) (Miyazono and Heldin, 1989; Ackland and Peters, 1999). TFT inhibits thymidylate synthase (TS) and is incorporated into the DNA (Eckstein *et al*, 1994; Emura *et al*, 2004d; Temmink *et al*, 2005). The inhibitor TPI is able to inhibit angiogenesis and metastasis by itself (Matsushita *et al*, 1999; Fukushima *et al*, 2000; Takao *et al*, 2000). It was previously shown *in vivo* that TPI in a ratio of 1 : 0.5 (TFT/TPI) was required to exert an *in vivo* activity (Emura *et al*, 2004c; Emura *et al*, 2005). However, *in vitro* TPI did not enhance the effect of TFT, also not in TP overexpressing cancer cells (De Bruin *et al*, 2003; Temmink *et al*, 2005). TAS-102 is currently undergoing clinical trials as an oral combination drug given in different schedules. In contrast to TAS-102, capecitabine (Xeloda) requires TP to be activated to the active metabolite 5-fluorouracil (5FU). TP catalyses the last activation step from the metabolite 5′DFUR to 5FU. 5′DFUR acts similar to 5FU by inhibition of TS and incorporation into RNA and DNA, although its activation pathway may affect its final mechanisms of action (Pinedo and Peters, 1988; Van Triest and Peters, 1999; Peters *et al*, 2002). Capecitabine is currently widely used as an oral 5FU prodrug in the treatment of colorectal cancer (Van Cutsem *et al*, 2005; Walko and Lindley, 2005).

As a result of the discrepancies between early *in vivo* work on TAS-102 and the *in vitro* data, we choose the HFA to dissect the role of TP in the *in vivo* antitumour activity of TAS-102. For this purpose, we used a colon cancer cell line deficient in TP (Colo320) and its TP-transfected variant Colo320TP1. As this cell line was 1000-fold more sensitive to 5′DFUR, we included 5′DFUR and its prodrug capecitabine as control compounds known to require TP for activation. TPI was included in the therapy experiment, in which not only growth inhibition, but also induction of cell cycle delay and apoptosis were investigated.

## MATERIALS AND METHODS

### Materials

Dulbecco's Modified Eagle's Medium (DMEM) and HEPES buffer were purchased from Cambrex BioScience (Verviers, Belgium) and foetal bovine serum (FBS) from Greiner Bio-One (Frickenhausen, Germany). TFT and TPI were synthesised and provided by Taiho Pharmaceuticals Co. (Tokushima, Japan). Capecitabine and 5′DFUR were provided by Roche Pharmaceuticals. 3-[4,5-Dimethylthiazol-2-yl]-2,5-diphenyl tetrazolium bromide (MTT) and propidium iodide (PI) were purchased from Sigma-Aldrich Chemicals (Zwijndrecht, The Netherlands). Bovine serum albumin (BSA) was obtained from Merck (Darmstadt, Germany). The biocompatible modified polyvinylidene difluoride (mPVDF) hollow fibres (CellMax^®^ Implant Membranes) with a *M*_r_ 500 kDa cutoff and 1.0 mm inner diameter were purchased from Spectrum Laboratories Inc. (Breda, The Netherlands). All other chemicals were of analytical grade and commercially available.

### Cell culture

The adherent *Mycoplasma*-free human colorectal cancer cell lines Colo320 (ATCC Nr: CCL-220) and Colo320TP1 were used for the experiments discussed in this paper. Colo320TP1 is a stable TP transfectant derived from Colo320 (low TP) and has increased expression of TP (De Bruin *et al*, 2003). Cells were grown in DMEM culture medium supplemented with 20 mM HEPES buffer and 10% heat-inactivated FBS (without antibiotics) and incubated at 37°C in a 5% CO_2_ humidified atmosphere. The cell lines have comparable growth rates with a doubling time of about 23 h.

### Preparation and *in vivo* implantation of the hollow fibres

The hollow fibre procedures were based on those of Hollingshead *et al* (1995). For the HFA immunocompetent host animals were used to test the human cancer cell lines, which were well tolerated, as was observed with various human cell lines in initial experiments. We did not observe any evidence of an immune reaction in the period that the fibres were present in the mice. Therefore, we proceeded with both Colo320 variants in immunocompetent BALB/c mice.

Before fibre implantation into the animals, we performed *in vitro* and *in vivo* experiments for optimisation of growth of Colo320 and Colo320TP1 cells in the fibres to determine the linearity in growth. The optimal cell suspension loading density was 0.5–1 × 10^6^ cells ml^−1^. The cells were harvested by a standard trypsinisation procedure and resuspended at the desired cell density (7.5 × 10^5^ cells ml^−1^). The cell suspension was flushed into the hollow fibres, whereafter they were heat-sealed and cut at 2 cm intervals. The fibres were incubated in DMEM medium in six-well plates 24 h prior to surgical implantation in immunocompetent 6- to 8-week-old female pure strain BALB/c mice (Harlan, Horst, The Netherlands). Three fibres per cell line were implanted s.c. with Colo320 at one flank and Colo320TP1 at the other flank of the mice. This ensured that both cell lines would undergo the same exposure reducing interindividual variation. The mice were anesthesised by isoflurane inhalation (day 0), and the incisions were closed using a stapling device. Separate *in vitro* control fibres were also prepared, and were incubated in DMEM medium during the experiment (10 days).

### Drug administration and retrieval of the cancer cells from the hollow fibres

The animals were kept in cages in an air-conditioned room with alternating cycles of light and dark and had access to food and autoclaved water *ad libitum*. The protocol was approved by the local Animal Ethical Committee in accordance with the UKCCR guidelines. The mice were treated on day 3 with the different fluoropyrimidines at their MTD by oral administration. We used 30 mice divided into six experimental groups: (1) control (saline); (2) TAS-102 (150 mg kg^−1^, q1dx5, based on TFT dose); (3) TFT (150 mg kg^−1^, q1dx5); (4) 5′DFUR (400 mg kg^−1^, on d1 and d5) + TPI (75 mg kg^−1^, q1dx5); (5) 5′DFUR (400 mg kg^−1^, on d1 and d5); (6) capecitabine (539 mg kg^−1^, q1dx5). The mice were killed by CO_2_ inhalation at day 10 and the fibres were excised from the mice. Excess host tissue was removed and the fibres were transferred into prewarmed DMEM medium and incubated for at least 1 h, whereafter the cells were retrieved from the (*in vitro* control) fibres for pharmacodynamic analysis. Per experimental group at least two fibres (bearing either Colo320 or Colo320TP1 cells) per animal (up to a maximum of six fibres) were used for assaying growth, cell cycle distribution or apoptosis induction. So in each mouse three different parameters could be assessed.

### Assessment of cytotoxicity

The ‘stable end point’ modified MTT assay (Alley *et al*, 1988; Xu *et al*, 1998) was used to determine cytotoxicity of the cancer cells to the fluoropyrimidine drugs after isolation of the fibres. The samples were stained and rinsed as described by the manufacturer. Briefly, the fibres were placed into 3 ml of fresh, prewarmed DMEM medium in six-well plates and allowed to equilibrate for 30 min at 37°C. Prewarmed DMEM (1.5 ml) containing 1 mg MTT/ml was added to each well. After a 4 h incubation at 37°C in a 5% CO_2_ humidified atmosphere the culture medium was aspirated and the fibres were washed with normal saline containing 2.5% protamine sulphate (w/v). The fibres were stored O/N at 4°C. The solution was aspirated and the samples were washed again for an additional 4 h at 4°C. Each fibre was wiped with a gauze to remove stained debris on the outside of the fibre, and transferred to 24-well plates 1 fibre well^−1^). The fibres were cut in half and allowed to dry O/N at RT. The produced formazan crystals were dissolved in dimethylsulphoxide (DMSO; 250 *μ*l well^−1^) and incubated for 4 h at RT on a rotating platform while protected from the light. Aliquots of 150 *μ*l of extracted solution were transferred to individual wells in 96-well plates. Absorbance (OD) was measured at 550 nm using a spectrophotometer (Spectra Fluor, Tecan, Salzburg, Austria). Net cell growth was calculated using the formula ((mean OD_d10_−mean OD_d0_)/(mean OD_d0_)) × 100%.

### Flow cytometry analysis

The cancer cells were retrieved from the fibres and prepared for gated flow cytometric analysis to measure cell cycle distribution of the cell population, as described previously (Cloos *et al*, 2002). Briefly, the cells were washed with saline/1% BSA solution and fixed with 70% ethanol (30 min on ice). After centrifugation, the pellet was resuspended in 400 *μ*l hypotonic PI solution (0.5 mg ml^−1^ RNase, 50 *μ*g ml^−1^ PI, 1 mg ml^−1^ sodium citrate, 1 *μ*l ml^−1^ Triton X-100 in saline) and incubated on ice (in the dark) for at least 15 min. Thereafter, the percentage of cells in the different cell cycle phases (G1, S, G2 M) was measured using a FACScan Flow Cytometer (Becton Dickinson Immunocytometry Systems, San Jose, CA, USA). For each measurement 25 000 cells were counted and each sample was assayed in duplicate. For calculation of the cell cycle distribution the Becton Dickinson's CellQuest software was used. The total number of cells in these fractions was set at 100%.

### Apoptosis analysis

The terminal deoxynucleotidyl transferase (TdT)-mediated dNTP labelling method was used for the detection of cells undergoing apoptosis (see also Bentz *et al*, 2004). We used the TdT-DNA-Fragment End Labeling Kit (FragEL^TM^; Calbiochem, Oncogene Research Products, Cambridge, MA, USA). In this method, TdT binds to exposed 3′-OH ends of DNA fragments generated in apoptotic cells in order to add biotin-(un)labelled dNTPs, which are detected using a streptavidin-horseradish peroxidase conjugate. The staining of the cells was performed according to the manufacturer's recommended procedure. Cells stained positive with 3′-3′-diaminobenzidine (DAB) produce a brownish colour, whereas nonreactive cells were counterstained with methylgreen. Actinomycin D-treated HL60 cells were included in the kit and served as positive controls whereas negative controls were untreated cells grown in tissue culture flasks. Using light microscopy, 1000 cells were counted twice for positive/negative staining on randomly selected areas on the glass slide. The Apoptosis Enrichment Factor was calculated as ((%) positive staining cells treated)/((%) positive staining cells control). Cells were defined as apoptotic when the (major part of) nuclear area was percentage-wise DAB-labelled.

### Statistics

The (un)paired Student's *t*-test was used for statistical evaluation of the results. Changes were considered to be significant when *P*<0.05.

## RESULTS

### Evaluation of hollow fibre model

The hollow fibres were quite well tolerated by the immunocompetent mice. The chemotherapeutic treatments did not affect the conditions of the mice beyond acceptable limits. The average body weight loss of the mice on day 10 was not more than 11% compared to the start of the treatment (day 3). We observed blood vessel formation around the implanted fibres after retrieval from the animals, and macroscopically a slight reduction in blood vessel formation was detected in the TPI treated animals, although not significantly. The antiangiogenic TPI is able to suppress angiogenesis in cells transfected with TP (Matsushita *et al*, 1999).

**Figure 1:**
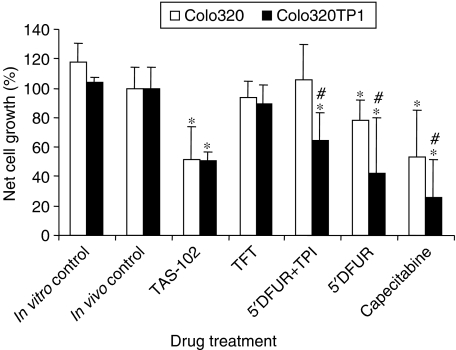
Growth inhibition of Colo320 and Colo320TP1 cells in the hollow fibres implanted s.c. in mice. The mice were treated with the fluoropyrimidines as described in Materials and methods. Four to five fibres per cell line implanted in different mice were assayed. Values are means±s.e. *In vitro* control: fibres were incubated in DMEM culture medium. Compared to *in vivo* fibre control: ^*^P<0.05; to Colo320: ^#^*P*<0.05.

After the treatment period cytotoxicity was assessed using the ‘stable end point’ modified MTT assay. Compared to the control fibres, TAS-102 treatment produced a significant reduction in net growth of about 50% for both Colo320 and Colo320TP1 (*P*<0.01) (Figure 1). Omission of TPI from the TAS-102 formulation completely abolished the antitumour effect of TAS-102; TFT alone was ineffective, underlining the importance of systemic inhibition of TP to increase bioavailability of TFT. The animals were also treated with capecitabine and its intermediate 5′DFUR to demonstrate the essential role of the presence of sufficient cellular TP activity, and to differentiate between the systemic and cellular effect of TPI. Both drugs were less active in Colo320 cells compared to Colo320TP1 cells (*P*<0.01), and addition of TPI completely abolished the activity of 5′DFUR in Colo320 cells.

### Cell cycle phase distribution

**Figure 2:**
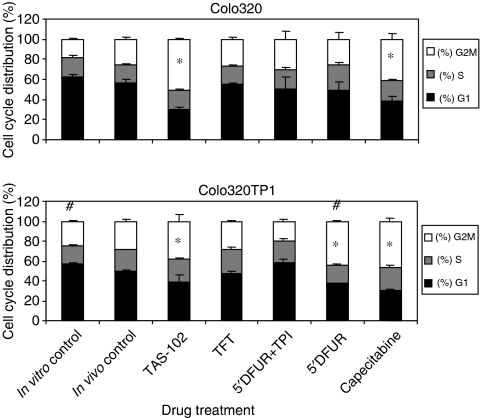
Cell cycle distribution for Colo320 and Colo320TP1 after retrieval from the hollow fibres. Per cell line three to five fibres from different mice were assayed using flow cytometry analysis. Values are means (%)±s.e.m. Total viable cell population was set at 100%. *In vitro* control: fibres were incubated in DMEM culture medium. (%) G2M compared to *in vivo* fibre control: ^*^P<0.05; to Colo320: ^#^*P*<0.05.

**Figure 3:**
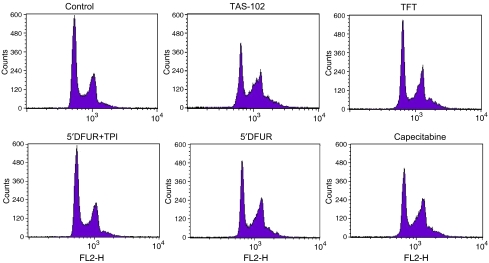
Representative DNA histograms to determine cell cycle distribution. The mice were treated with the fluoropyrimidines as described in Materials and Methods. After retrieval, the fibres were assayed to measure the percentage of nonapoptotic Colo320TP1 cells in the G1-, S- and G2M-phases. A clear G2M-arrest was observed for mice treated with TAS-102, 5′DFUR or Capecitabine.

From all experimental groups cell suspensions were retrieved from fibres of different animals to be assayed for cell cycle phase distribution (Figure 2). Representative DNA histograms are depicted in Figure 3. The cell cycle distribution in the *in vivo* control fibres was not significantly different from the *in vitro* control fibres, although a tendency for increased G2M-phase was found. In all drug-treated animals the S-phase population hardly changed compared to the *in vivo* control fibres. In contrast, TAS-102 treatment resulted in a clear G2M-phase arrest for both cell lines (*P*<0.05). However, omission of TPI from the TAS-102 formulation completely neutralised this effect. Treatment of animals with 5′DFUR or capecitabine alone resulted in Colo320TP1 a strong decrease of G1-population and increased G2M-population (*P*<0.05). Capecitabine treatment also resulted in a G2M-phase arrest in Colo320 cells, but less than in Colo320TP1 cells. TPI treatment prevented the induction of G2M-phase arrest in Colo320TP1 cells. These data all underline the importance of cellular activation of 5′DFUR.

### Induction of apoptosis

**Figure 4:**
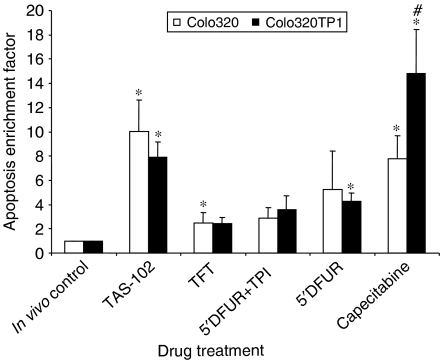
Induction of apoptosis in Colo320 and Colo320TP1 cells grown in hollow fibres. Per cell line three to five fibres from different mice were assayed for apoptosis. Values are means±s.e.m. and were expressed as (%) apoptosis treated/(%) apoptosis control (was set at 1). Compared to fibre control: ^*^P<0.05; to Colo320: ^#^*P*<0.05.

Retrieval of the cells from the implanted fibres enabled to evaluate apoptosis induction (Figure 4). The fibres retrieved from the control animals contained on average <3% apoptotic cells. TAS-102 treatment induced apoptosis eight- to 10-fold (*P*<0.05) both in Colo320TP1 and Colo320. However, omission of TPI reduced the apoptosis induction by TFT almost to control levels. Capecitabine treatment resulted in the highest extent of apoptosis in Colo320TP1 cells, which was significantly reduced in the low TP-expressing Colo320 cells (*P*=0.02). Treatment with 5′DFUR caused a moderate apoptosis induction (about four-fold), which was reduced by co-treatment with TPI.

## DISCUSSION

This paper shows that the HFA can be used conveniently to demonstrate the importance of TP in the activity or resistance to various oral fluoropyrimidine formulations. The TAS-102 formulation, consisting of TFT and TPI, was inactive without TPI administration. However, in *in vitro* studies the presence of TPI did not enhance the activity of TFT, underlining the importance of suitable *in vivo* systems to investigate this formulation. The intermediate of capecitabine, 5′DFUR, was inactive in cells with low TP expression.

The HFA is currently used by the NCI in the US for secondary drug screening and saves time and labour compared to the classical tumour implantation experiments. Recently, several papers have been published demonstrating that the HFA is also suited for short-term *in vivo* pharmacodynamic studies (Hall *et al*, 2000; Leong *et al*, 2004; Suggitt *et al*, 2004; Suggitt and Bibby, 2005), thereby using immunocompetent mice for the growth of human cells, without problems such as infiltrating immune cells in this period. Our results showed that the HFA is excellently suitable for studying several pharmacodynamic end points in cancer cells when treated *in vivo* with fluoropyrimidines.

The advantage of the HFA for our studies was the possibility to investigate the *in vivo* role of TPI in the formulation TAS-102. In previous *in vitro* studies, we did not observe an advantage of TPI in a combination with TFT, even not in cancer cells with high TP expression (De Bruin *et al*, 2003; Temmink *et al*, 2005). This was despite previous observations that TPI was essential to observe an antitumour effect of TFT (Fukushima *et al*, 2000; Emura *et al*, 2004c), by increasing the bioavailability of TFT (Emura *et al*, 2005). TFT has been evaluated in early clinical trials as a single agent (Ansfield and Ramirez, 1971), but proved to be ineffective in colorectal cancer patients, probably because of its rapid and extensive degradation by TP and subsequent elimination from the body. TAS-102 was developed to bypass these limitations, while it is also active in 5FU-resistant tumours (Emura *et al*, 2004a, d). The presence of TPI has the additional advantage that it induces an antiangiogenic effect (Matsushita *et al*, 1999). Currently, three phase I trials are being performed with TAS-102, but no efficacy data are available.

In contrast to *in vitro* studies, but similar to the early clinical studies (Ansfield and Ramirez, 1971), TFT alone was highly ineffective, even against Colo320, which has a very low TP expression and is very sensitive to the drug *in vitro*. TFT apparently does not reach the cancer cells in order to exert any activity. However, TPI significantly improved TFT-mediated cytotoxicity to both low TP expressing Colo320 and even the high TP expressing Colo320TP1 cells. Flow cytometric analysis showed that TAS-102 treatment induced a G2M-phase arrest in the cell populations, and induced apoptosis in the p53 mutated Colo320 cells, probably as a result of the induced cell cycle delay. Matsushita *et al* (1999) previously showed that treatment with TPI alone can increase apoptosis induction in tumours of the high TP expressing human KB epidermoid carcinoma. However, the main action of TPI seemed to consist of the increase in the bioavailability in the hollow fibres, enabling to activate TFT similarly in Colo320 and Colo320TP1 cells, because these two cell lines showed similar cell cycle disturbances and apoptosis. In addition, TPI can also increase TdR concentrations in plasma (Emura *et al*, 2004b), which can prevent breakdown of TFT, because TdR can compete with TFT in TP-mediated phosphorolysis. *In vitro* we did not find evidence that this TdR accumulation would negatively affect TFT-induced cytotoxicity.

As a positive control to differentiate between the *in vivo* systemic and cellular role of TP in the activity of fluoropyrimidines, we investigated the effect of capecitabine and its intermediate 5′DFUR. Both drugs were less effective in the wild-type Colo320, which was accompanied by less pronounced cell cycle effects and apoptosis. However, both 5′DFUR and capecitabine showed activity in the TP deficient Colo320, which is not in line with the large difference found *in vitro* (De Bruin *et al*, 2003; Temmink *et al*, 2005). The partial protection by TPI of 5′DFUR′s cytotoxicity against Colo320 demonstrates that 5′DFUR is systemically converted to 5FU, contradicting a selective cellular specificity of 5′DFUR. Although TPI protected the cells from 5′DFUR, this was only partial as can clearly be seen from the apoptotic effect. This demonstrated that 5′DFUR activation is not only dependent on TP, as anticipated (De Bruin *et al*, 2006), but also that another phosphorylase, presumably uridine phosphorylase, plays a role. In contrast, a high TP expression might theoretically inactivate TFT, but this does not seem to happen. *In vitro* TFT is cytotoxic against high TP expressing cells, and here we show that TAS-102 is also equally active against high and low TP expressing cells. This means that for *in vivo* and clinical application high TP levels in the tumour are not necessarily a negative parameter, while TAS-102 is active against low TP expressing cells, in contrast to 5′DFUR, which needs high TP for selectivity.

An additional advantage of TAS-102 will be the potential antiangiogenic effect of TPI. Phillips *et al* (1998) reported that efficiency of drug delivery and the subsequent effects on chemosensitivity of the encapsulated tumour cells in the hollow fibre can be enhanced due to increased angiogenesis. After at least 6-days, the mice carrying hollow fibres s.c. may develop extensive vascular networks. For long-term angiogenesis studies the postimplantation time must be extended to determine the effect of a potentially antiangiogenic treatment. Regarding these studies it may be interesting to investigate TAS-102 with respect to inhibition of blood vessel formation in tumours since TPI also inhibits PD-ECGF, which is considered to be proangiogenic (Haraguchi *et al*, 1994). We observed after 10-days treatment with TAS-102 that TPI acts as an antiangiogenic compound (as previously shown (Matsushita *et al*, 1999; Emura *et al*, 2005)) and is likely to be related to the suppression of metastasis (Takao *et al*, 2000; Sato *et al*, 2003). With respect to these findings it will be interesting to combine TAS-102 with other agents that counteract blood vessel formation such as bevacizumab, which targets the proangiogenic VEGF. Another possibility is to combine TAS-102 with other novel agents in the future, such as the EGFR-targeting cetuximab or erlotinib.

In conclusion, TAS-102 was effective in the HFA, which was not only shown by a clear cytotoxicity, but also by the change in several pharmacodynamic end points *in vivo*, such as cell cycle distribution and apoptosis. These parameters could be studied conveniently *in vivo*, and provide an excellent basis to study the antitumour activity of potential TAS-102 applications using the HFA, such as in combination with targeted agents.
